# Metal–Ligand Cooperation with Thiols as Transient Cooperative Ligands: Acceleration and Inhibition Effects in (De)Hydrogenation Reactions

**DOI:** 10.1021/acs.accounts.4c00198

**Published:** 2024-06-04

**Authors:** Jie Luo, Michael Montag, David Milstein

**Affiliations:** Department of Molecular Chemistry and Materials Science, Weizmann Institute of Science, Rehovot 7610001, Israel

## Abstract

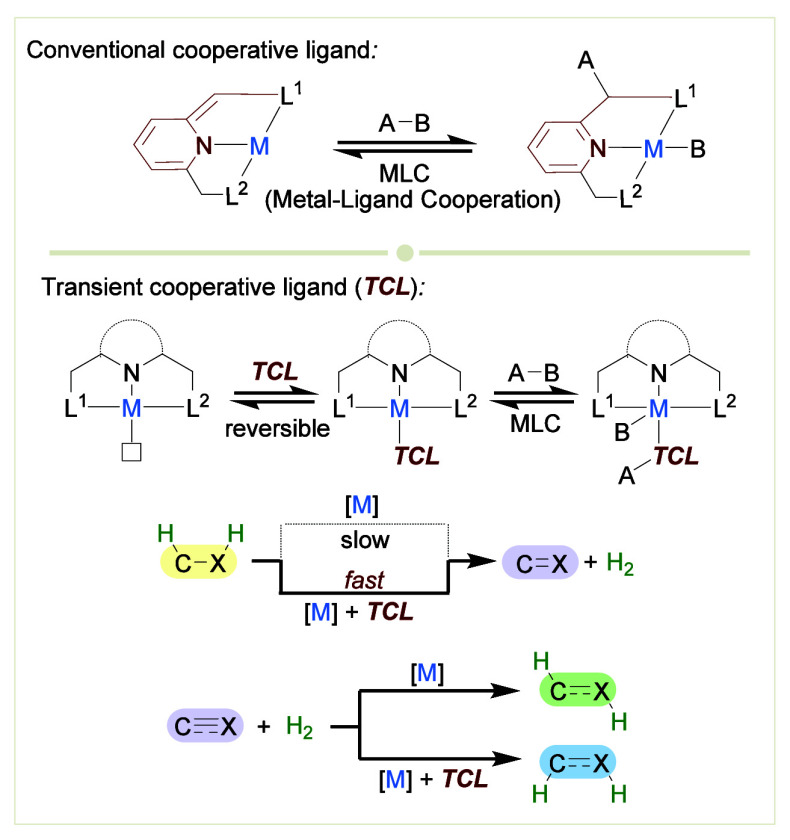

Over the past two decades, we have developed a series of pincer-type transition metal complexes capable of activating strong covalent bonds through a mode of reactivity known as metal–ligand cooperation (MLC). In such systems, an incoming substrate molecule simultaneously interacts with both the metal center and ligand backbone, with one part of the molecule reacting at the metal center and another part at the ligand. The majority of these complexes feature pincer ligands with a pyridine core, and undergo MLC through reversible dearomatization/aromatization of this pyridine moiety. This MLC platform has enabled us to perform a variety of catalytic dehydrogenation, hydrogenation, and related reactions, with high efficiency and selectivity under relatively mild conditions.

In a typical catalytic complex that operates through MLC, the cooperative ligand remains coordinated to the metal center throughout the entire catalytic process, and this complex is the only catalytic species involved in the reaction. As part of our ongoing efforts to develop new catalytic systems featuring MLC, we have recently introduced the concept of *transient cooperative ligand* (TCL), i.e., a ligand that is capable of MLC when coordinated to a metal center, but the coordination of which is reversible rather than permanent. We have thus far employed thiol(ate)s as TCLs, in conjunction with an acridanide-based ruthenium(II)-pincer catalyst, and this has resulted in remarkable acceleration and inhibition effects in various hydrogenation and dehydrogenation reactions. A cooperative thiol(ate) ligand can be installed *in situ* by the simple addition of an appropriate thiol in an amount equivalent to the catalyst, and this has been repeatedly shown to enable efficient bond activation by MLC without the need for other additives, such as base. The use of an ancillary thiol ligand that is not fixed to the pincer backbone allows the catalytic system to benefit from a high degree of tunability, easily implemented by varying the added thiol. Importantly, thiols are coordinatively labile enough under typical catalytic conditions to leave a meaningful portion of the catalyst in its original unsaturated form, thereby allowing it to carry out its own characteristic catalytic activity. This generates two coexisting catalyst populations—one that contains a thiol(ate) ligand and another that does not—and this may lead to different catalytic outcomes, namely, enhancement of the original catalytic activity, inhibition of this activity, or the occurrence of diverging reactivities within the same catalytic reaction mixture. These thiol effects have enabled us to achieve a series of unique transformations, such as thiol-accelerated base-free aqueous methanol reforming, controlled stereodivergent semihydrogenation of alkynes using thiol as a reversible catalyst inhibitor, and hydrogenative perdeuteration of C=C bonds without using D_2_, enabled by a combination of thiol-induced acceleration and inhibition. We have also successfully realized the unprecedented formation of thioesters through dehydrogenative coupling of alcohols and thiols, as well as the hydrogenation of organosulfur compounds, wherein the cooperative thiol serves as a reactant or product. In this *Account*, we present an overview of the TCL concept and its various applications using thiols.

## Key References

- LuoJ.; RauchM.; AvramL.; Diskin-PosnerY.; ShmulG.; Ben-DavidY.; MilsteinD.Formation of Thioesters by Dehydrogenative Coupling of Thiols and Alcohols with H_2_ Evolution. Nat. Catal.2020, 3, 887–892. Acridanide-based ruthenium thiol(ate) complexes were isolated for the first time and demonstrated to reversibly activate H_2_*.*^1^
- LuoJ.; KarS.; RauchM.; MontagM.; Ben-DavidY.; MilsteinD.Efficient Base-Free Aqueous Reforming of Methanol Homogeneously Catalyzed by Ruthenium Exhibiting a Remarkable Acceleration by Added Catalytic Thiol. J. Am. Chem. Soc.2021, 143, 17284–17291.34617436 10.1021/jacs.1c09007PMC8532156 Addition of a catalytic amount of thiol resulted in remarkable acceleration of methanol reforming catalyzed by a ruthenium pincer complex.^2^
- LuoJ.; LiangY.; MontagM.; Diskin-PosnerY.; AvramL.; MilsteinD.Controlled Selectivity through Reversible Inhibition of the Catalyst: Stereodivergent Semihydrogenation of Alkynes. J. Am. Chem. Soc.2022, 144, 13266–13275.35839274 10.1021/jacs.2c04233PMC9374179 Thiol-induced inhibition was observed to control the selectivity of alkyne semihydrogenation catalyzed by a ruthenium pincer complex.^3^
- LuoJ.; LuL.; MontagM.; LiangY.; MilsteinD.Hydrogenative Alkene Perdeuteration Aided by a Transient Cooperative Ligand. Nat. Chem.2023, 15, 1384–1390.37667011 10.1038/s41557-023-01313-y The concept of transient cooperative ligand was introduced, encompassing both acceleration and inhibition effects, and enabling the Ru-catalyzed hydrogenative perdeuteration of C=C bonds without the use of D_2_.^4^

## Introduction

**Scheme 1:**
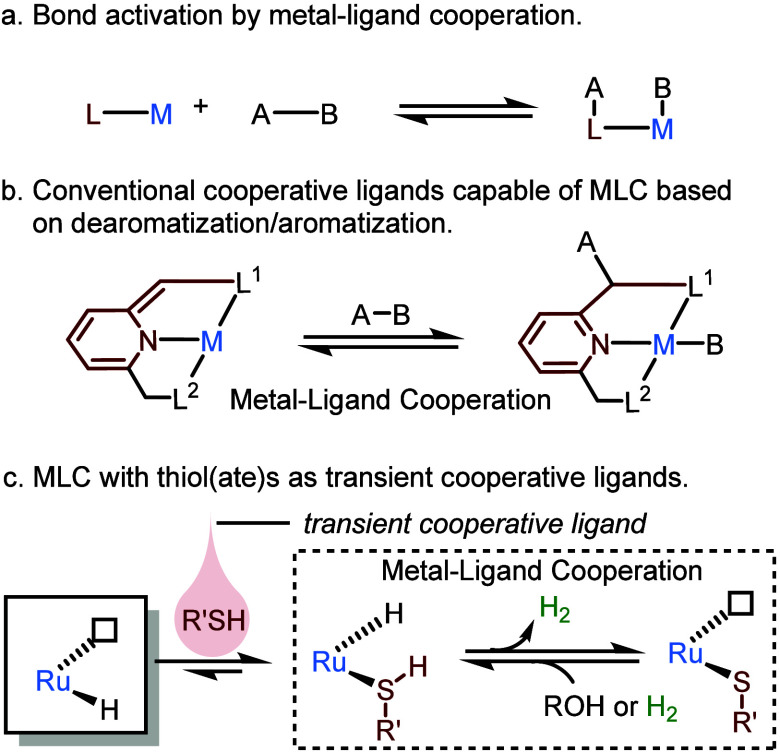
Conventional Metal–Ligand Cooperation through Dearomatization/Aromatization and Metal–Ligand Cooperation with Thiol(ate)s as Transient Cooperative Ligands

Over the past few decades, significant advancements have been made in catalytic bond activation by metal complexes, leading to the development of new reactions for organic synthesis and sustainable processes.^5−9^ Traditionally, such reactions would occur solely at the metal center of a given catalytic complex. However, recent examples of transition metal complexes featuring cooperative ligands, capable of what is known as metal–ligand cooperation (MLC), have drawn increasing attention to such systems as promoters of chemical bond activation (Scheme 1a).^10−12^ In these complexes, the ligands surrounding the metal center do not only modulate its properties, but also actively participate in bond activation. This has given rise to unique catalytic attributes, which have significantly impacted homogeneous catalysis.

We have previously discovered a series of metal complexes bearing pyridine-based pincer ligands, which can activate strong covalent bonds through MLC involving reversible dearomatization/aromatization of the pyridine core (Scheme 1b).^13−15^ These systems are typically preactivated by base-induced deprotonation of the ligand side arm to generate a dearomatized form of the pincer complex, wherein one cooperative site that participates in bond activation resides at that side arm, whereas the second site is at the metal center. Various inert bonds, including H–H, C–H, O–H, N–H, and B–H, could be heterolytically cleaved and added across the metal–ligand framework through synergistic action of both sites. Relying on this cooperative process, we have developed an assortment of unprecedented, efficient and environmentally friendly dehydrogenative coupling reactions involving the release of hydrogen gas, as well as the reverse, highly atom-economical hydrogenation reactions.^16^

**Scheme 2:**
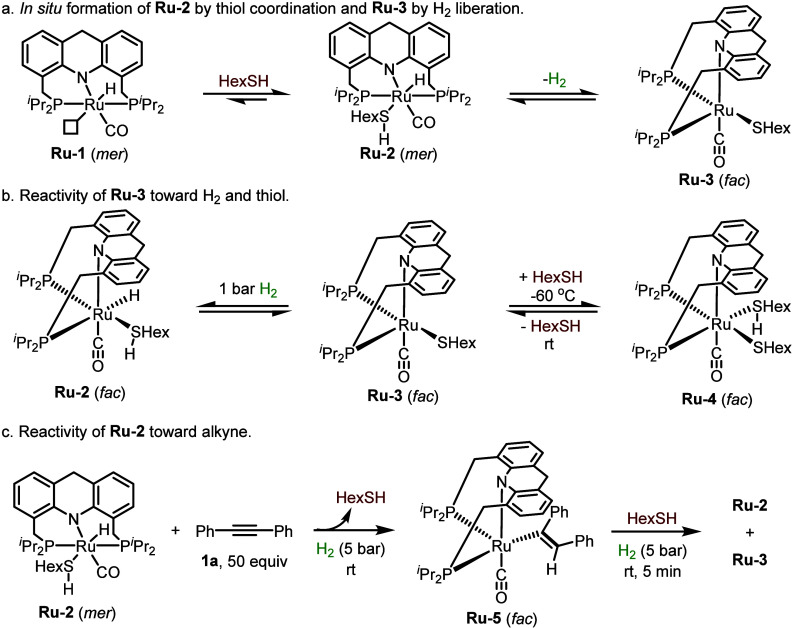
Reversible Activation of H_2_ with Thiol(ate) as a Transient Cooperative Ligand and Related Reactions

In this *Account*, we overview our recent investigations on a novel MLC strategy that utilizes *transient cooperative ligands* (TCLs; Scheme 1c). A TCL is a ligand that coordinates reversibly to a metal center, and is capable of MLC when coordinated.^17,18^ In our case, we applied thiol(ate)s as TCLs, in conjunction with an acridanide-based ruthenium(II)-pincer catalyst, **Ru-1** (Scheme 2). A thiol(ate) TCL can be formed *in situ* by simply adding an appropriate thiol to the catalytic complex. Importantly, the coordinative lability of these cooperative ligands enables them to dissociate from the metal center, thereby leaving a substantial portion of the original complex to carry out its own characteristic catalytic activity (e.g., catalysis involving the metal–hydride moiety of **Ru-1**). Therefore, adding a TCL establishes a dual catalytic system comprised of two distinct coexisting catalytic species that can simultaneously promote different reactions. In principle, these reactions can be further controlled through judicious choice of the externally added cooperative ligand. In the examples discussed below, the presence of TCLs has resulted in remarkable acceleration and inhibition effects in various hydrogenation and dehydrogenation processes. By recounting the unique features of TCLs, as they are reflected in the thiol(ate)-based systems, along with mechanistic insights, we aim to encourage the extension of the TCL concept to other catalytic reactions, and inspire the design of new catalytic systems involving TCLs.

## Reversible Activation of H_2_ by Metal–Ligand Cooperation with Thiol(ate)s as Transient Cooperative Ligands^1,3^

Metal–thiolate bonds are ubiquitous in nature and play critical roles in various enzymatic transformations, such as the heterolytic splitting of dihydrogen catalyzed by hydrogenases.^19^ A notable example is the active site of [NiFe] hydrogenase, where the metal-bonded thiolate, a cysteinate, facilitates the conversion of H_2_ into protons and electrons.^20^ The proton transfer capability of thiolates has been harnessed in several artificial hydrogenase mimics to facilitate the H_2_ evolution reaction,^21^ as well as in other bioinspired metal complexes used for hydrogen and oxygen activation.^22−26^ For example, Wang and co-workers showed that a trithiolato-diiron-hydride complex catalyzes H/D exchange between H_2_ and D_2_O through a process that involves reversible protonation of a terminal thiolate ligand.^26^

**Figure 1:**
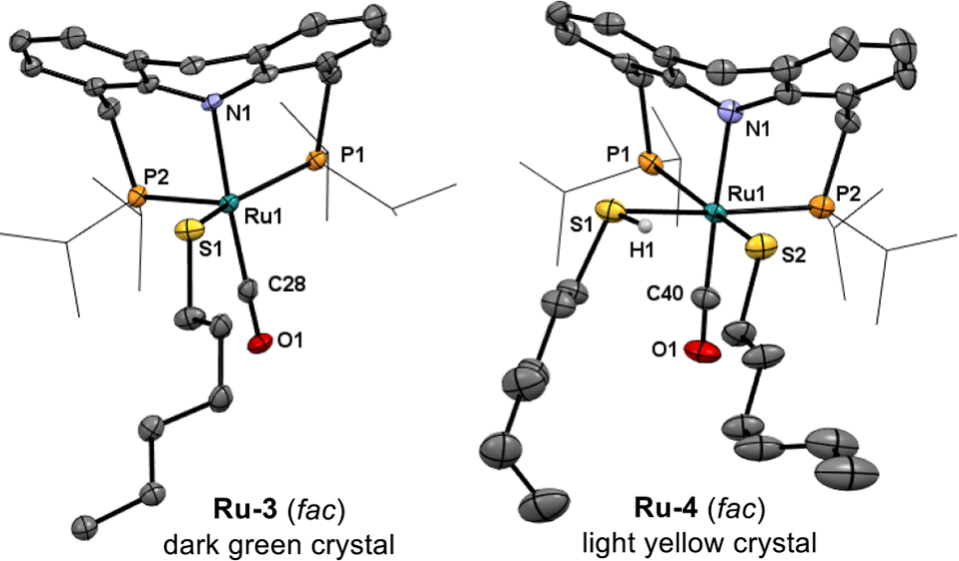
X-ray crystal structures of **Ru-3** and **Ru-4**.

We have recently reported that **Ru-1** reacts with 1 equiv of hexanethiol (HexSH) to quantitatively generate a hydrido-thiol ruthenium(II) complex, **Ru-2** (Scheme 2a).^1^ This complex can further convert into a ruthenium(II) thiolate complex, **Ru-3**, with concomitant release of one molecule of H_2_ per complex. The extrusion of H_2_ from **Ru-2**, which can take place even at room temperature, involves MLC, as the proton of the thiol S–H moiety couples with the ruthenium-bonded hydride. This intramolecular reaction was studied computationally using density functional theory (DFT), revealing a very low kinetic barrier, i.e., Δ*G*^‡^ = 11.7 kcal/mol (see below).^4^ The molecular structure of **Ru-3** was determined experimentally by X-ray crystallography, showing a *facial* (*fac*) conformation of the pincer ligand, with its central nitrogen donor and two flanking phosphorus donors in mutually *cis* positions (Figure 1). Interestingly, the transformation of **Ru-2** into **Ru-3** is reversible (Scheme 2b), and under 1 bar of H_2_, **Ru-3** converts into *fac-***Ru-2** at room temperature. The latter gradually isomerizes into the thermodynamically more stable *meridional* (*mer*) form of **Ru-2**. This reaction clearly demonstrates the ability of **Ru-3** to heterolytically split H_2_, facilitated by the cooperation between the ruthenium center and its thiolate ligand.

A noteworthy feature of **Ru-3** is that it can retain a vacant coordination site even in the presence of excess thiol. Only by lowering the solution temperature to −60 °C could we observe the coordination of a second molecule of thiol, leading to complex **Ru-4** (Scheme 2b), the structure of which was confirmed by X-ray crystallography (Figure 1). In solution, this complex is thermally unstable, quickly reverting to **Ru-3** and free thiol upon warming to room temperature. Thus, the coordinative lability of the thiol, driven by entropic effects, allows **Ru-3** to maintain its empty coordination site during catalysis, at room temperature or under heating. This vacancy, in turn, is crucial for enabling the metal center to accept a hydride ligand during (de)hydrogenation reactions (see below).

Thiol lability is also an important attribute of **Ru-2**. In analogy to **Ru-4**, the coordinated thiol in **Ru-2** can dissociate from the ruthenium center, even at room temperature, and be displaced by other ligands. For instance, when **Ru-2** was treated with excess diphenylacetylene (**1a**) at room temperature, the alkenyl ruthenium(II) species **Ru-5** immediately formed, with concomitant liberation of thiol (Scheme 2c).^3^ Complex **Ru-5** is, at least formally, the product of alkyne insertion into the Ru–H bond of **Ru-2**. The fact that the coordinatively saturated **Ru-2** easily reacts with an alkyne to give the thiol-free complex **Ru-5** indicates that the thiol ligand can readily dissociate from **Ru-2**, thereby leaving a vacant site to which **1a** can coordinate, and then react intramolecularly with the hydride. This underscores the notion that a TCL not only facilitates bond activation through MLC when it is coordinated to the metal center, but its ability to dissociate from this metal center also provides an opportunity for the original catalyst to carry out its own catalytic activity. Taken together, the unique properties of TCLs result in catalytic activities that are distinct from those of complexes containing conventional cooperative ligands, as demonstrated by the following examples.

## Acceleration Effects of Transient Cooperative Thiol Ligands in Base-Free Aqueous Methanol Reforming^2^

The production of H_2_*via* methanol reforming is of considerable interest due to the low cost, wide availability, and high hydrogen content of methanol. Conventional heterogeneous catalysts for this reaction typically require high temperatures and pressures, but homogeneous catalytic systems developed over the past decade can promote this reaction under much milder conditions, bringing us closer to a methanol-based economy.^27^ Thus far, the most successful of these systems have utilized pincer complexes capable of MLC, which significantly reduces the kinetic barriers associated with bond activation. However, an intrinsic drawback of these catalytic systems is the requirement for excess base. This is because typical catalysts involving MLC, which operate *via* deprotonation/protonation and dearomatization/aromatization, are poisoned by acidic species generated during methanol reforming, namely, formic acid and CO_2_, and these must be scavenged by base to ensure efficient turnover.^28^

**Scheme 3:**
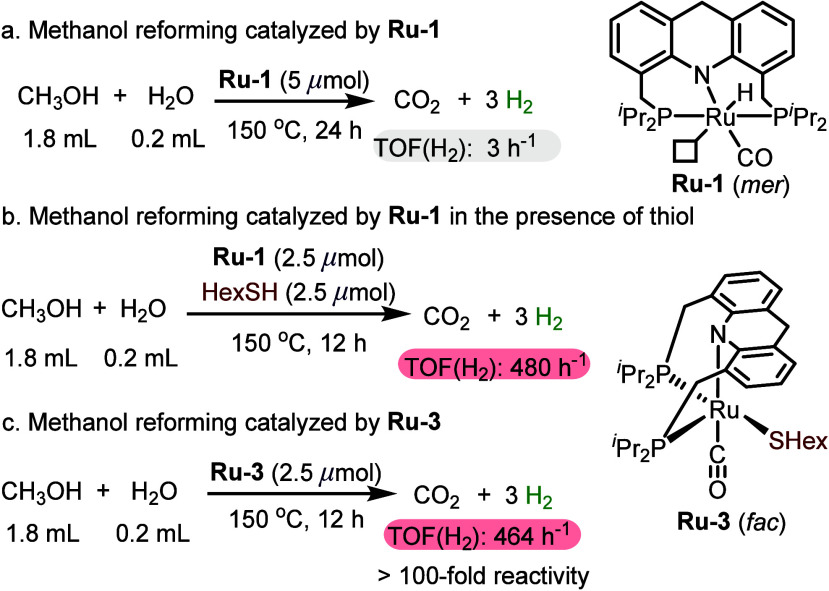
Thiol-Accelerated Methanol Reforming

While investigating base-free aqueous methanol reforming by **Ru-1**, we serendipitously discovered that a catalytic amount of thiol can greatly accelerate this reaction. Initially, **Ru-1** was found to inefficiently catalyze the reforming reaction, with an H_2_-based turnover frequency [TOF(H_2_)] of only 3 h^–1^ (Scheme 3a). However, adding HexSH to the reaction mixture containing **Ru-1**, in an amount equivalent to the catalyst, enhanced the rate of catalysis by over 2 orders of magnitude, leading to a TOF(H_2_) of 480 h^–1^ (Scheme 3b).^2^ As shown in Scheme 2a, **Ru-1** reacts with HexSH to eventually give the thiolate complex **Ru-3**, and the latter is therefore expected to form *in situ* in the methanol reforming system. Indeed, independently synthesized **Ru-3** was found to catalyze methanol reforming essentially as efficiently as the **Ru-1**/HexSH combination, exhibiting TOF(H_2_) = 464 h^–1^ (Scheme 3c).

**Figure 2:**
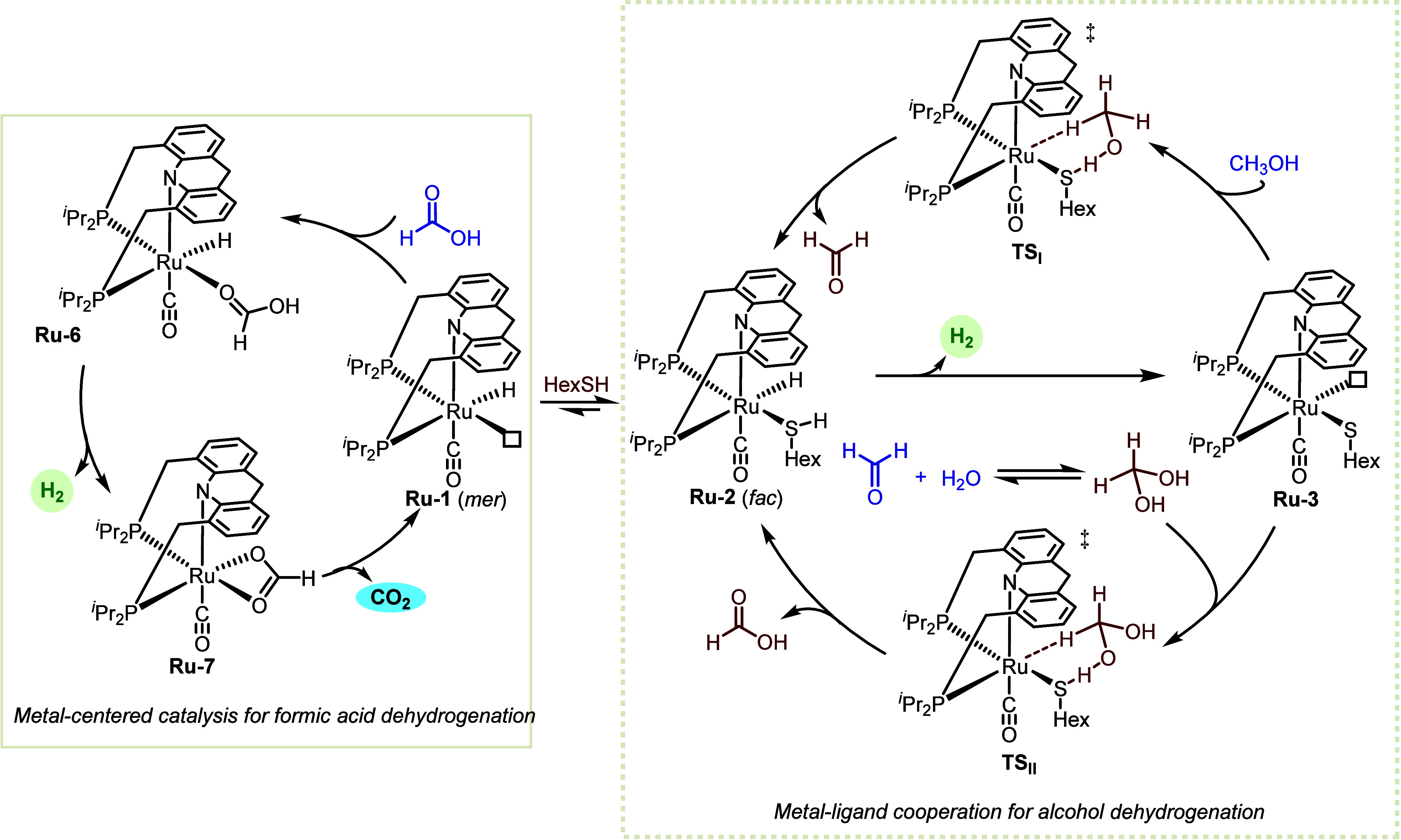
Proposed mechanism of aqueous methanol reforming by **Ru-1** in the presence of thiol.

According to our proposed catalytic mechanism, **Ru-3** initiates methanol reforming by dehydrogenating methanol into CH_2_O, and then dehydrogenating methanediol - the spontaneously generated hydrate of CH_2_O - into formic acid (Figure 2, right cycle). Our DFT data show that this occurs through outer-sphere transition states (**TS**_**I**_ and **TS**_**II**_), with the thiolate serving as a cooperative ligand that receives a proton from a hydroxyl group, whereas the ruthenium center abstracts a hydride from the C–H moiety. This reaction pathway, by which **Ru-3** transforms methanol and H_2_O into HCOOH and H_2_, was found to have an apparent kinetic barrier of 35.6 kcal/mol, which is substantially lower than the energy barrier calculated for **Ru-1** (≥43.4 kcal/mol). This thiol-induced decrease in the activation energy accounts for the observed acceleration effect upon addition of thiol to **Ru-1**, highlighting the role of MLC in the activation of chemical bonds.

**Scheme 4:**
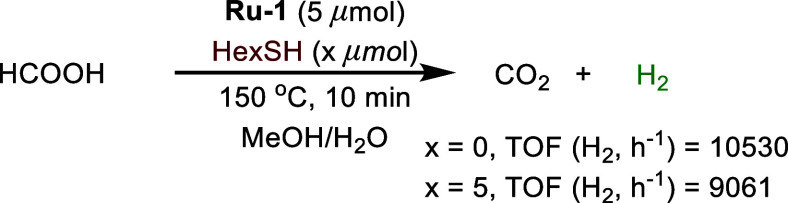
Dehydrogenation of Formic Acid by Ru-1

Interestingly, we found that dehydrogenation of formic acid, which is the final step of methanol reforming, and had been previously shown to be promoted by **Ru-1**,^29^ occurs more efficiently in the absence of thiol than in its presence [TOF(H_2_) of 10530 vs 9061 h^–1^, respectively; Scheme 4]. This suggests that thiol inhibits the dehydrogenation of formic acid, possibly by competing with it for coordination to the ruthenium center, and also indicates that **Ru-1** is the primary catalyst responsible for formic acid dehydrogenation within the methanol reforming system. Therefore, our experimental and computational evidence indicates that all three ruthenium complexes, i.e., **Ru-1** and its thiol(ate)-containing derivatives **Ru-2** and **Ru-3**, participate in the base-free aqueous methanol reforming process (Figure 2). This underscores the significance of the transient nature of the cooperative thiol(ate) ligand, which not only engages in methanol and methanediol activation when it is coordinated to the metal center of **Ru-3**, but can also dissociate from **Ru-2** to release **Ru-1** and enable it to perform formic acid dehydrogenation, thereby completing the entire reforming cycle.

**Figure 3:**
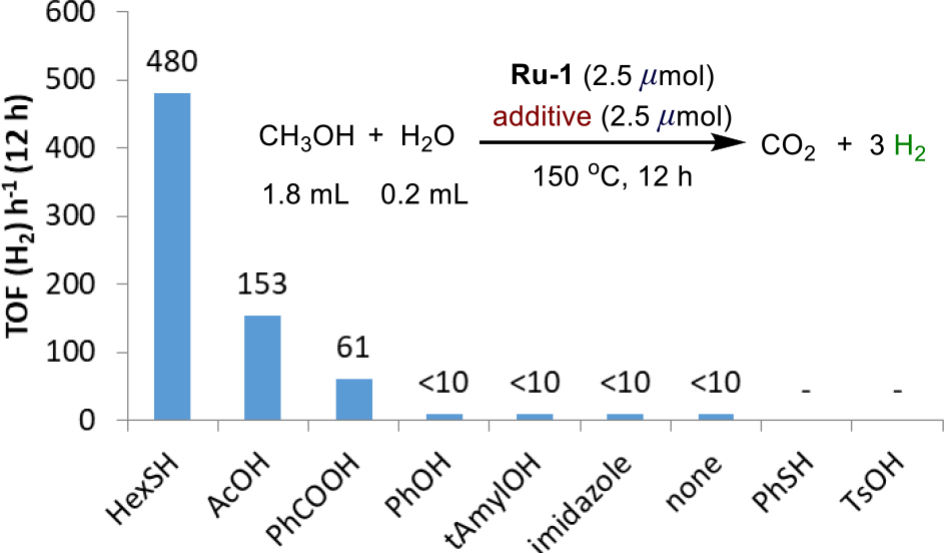
Effect of different additives on the methanol reforming activity of **Ru-1**.

It is important to stress that such MLC with a TCL is not restricted to thiol(ate) ligands. We have also explored combinations of **Ru-1** with other additives, and some, such as carboxylic acids, have also been found to enhance the methanol reforming activity of the ruthenium complex, albeit to a lesser extent than HexSH (Figure 3). In these instances, it is presumed that ruthenium carboxylate complexes are formed, which facilitate bond activation in a manner similar to **Ru-3**. It should be noted that carboxylates are frequently employed to facilitate C–H activation reactions.^30,31^

## Inhibition Effects of Transient Cooperative Ligands Enable Stereocontrolled Semihydrogenation of Alkynes^3^

**Scheme 5:**
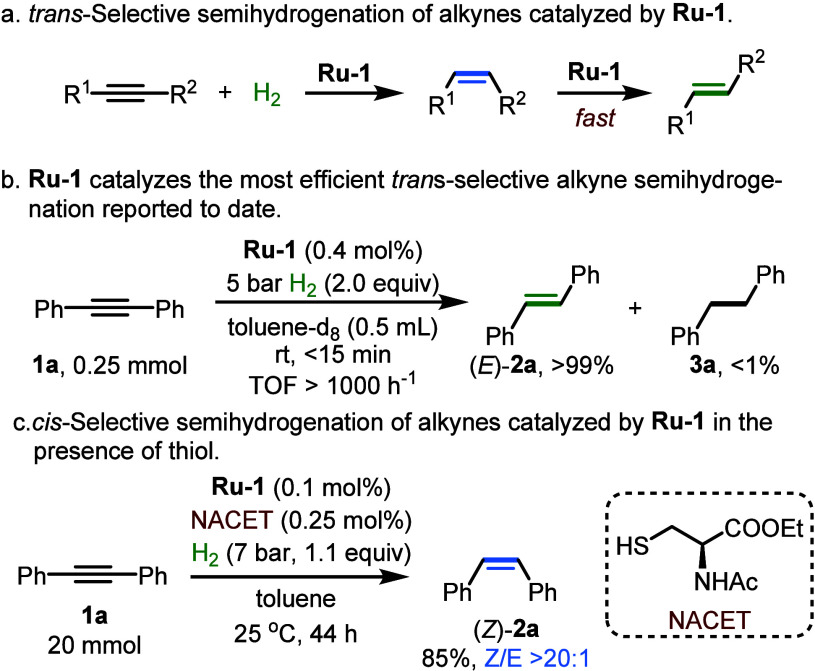
Alkyne Semihydrogenation with Thiol-Controlled Switchable Stereoselectivity

Catalytic semihydrogenation of internal alkynes is an attractive route to access different alkenes for small-scale laboratory synthesis, as well as large-scale industrial production.^32,33^ We have previously reported that **Ru-1** is a highly active catalyst for *trans*-semihydrogenation of alkynes, achieving this through *cis*-semihydrogenation followed by rapid Z/E isomerization of the alkene products (Scheme 5a).^3^ For instance, **1a** could be fully converted into *trans*-stilbene, (*E*)-**2a**, in less than 15 min at room temperature, corresponding to a TOF of over 1000 h^–1^, which represents the most efficient *trans*-selective alkyne semihydrogenation reported to date (Scheme 5b).

**Figure 4:**
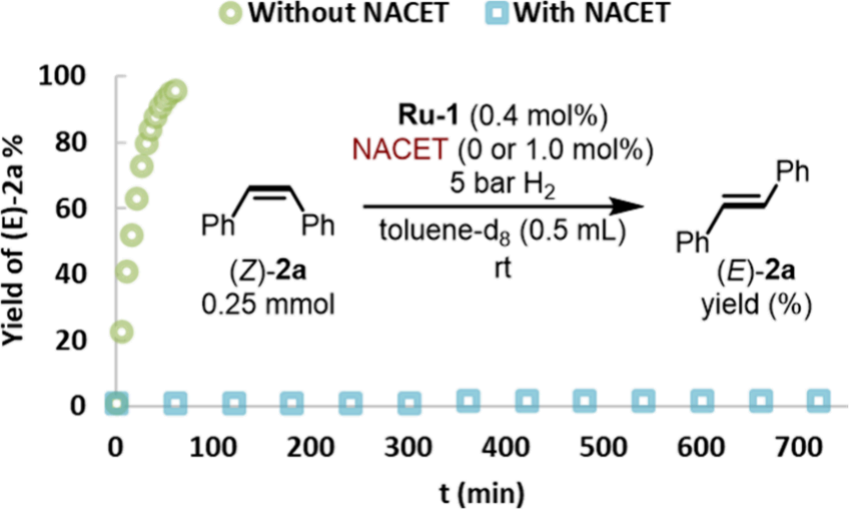
Control experiments for Z/E isomerization by **Ru-1** in the absence and presence of thiol (NACET, *N*-acetylcysteine ethyl ester).

Intriguingly, addition of thiols to the catalytic reaction mixture containing **Ru-1** and alkyne was found to invert the selectivity of semihydrogenation, from *trans* to *cis*. For example, employing *N*-acetylcysteine ethyl ester (NACET) selectively facilitated the *cis*-semihydrogenation of **1a**, with (*Z*)-**2a** becoming the major final product (Scheme 5c). Control experiments indicated that the isomerization rate of (*Z*)-**2a** drops by a factor of >500 in the presence of NACET, explaining the inverted stereoselectivity (Figure 4). Therefore, this thiol can serve as a highly reliable catalyst inhibitor,^34−36^ selectively preventing the metal center from interacting with the alkene product, and thus avoiding its isomerization. Mechanistic investigations indicated that the enhanced selectivity observed with NACET, compared to the other thiols examined in this study, can be attributed to both its acidity and its ability to reversibly chelate the ruthenium center.

**Figure 5:**
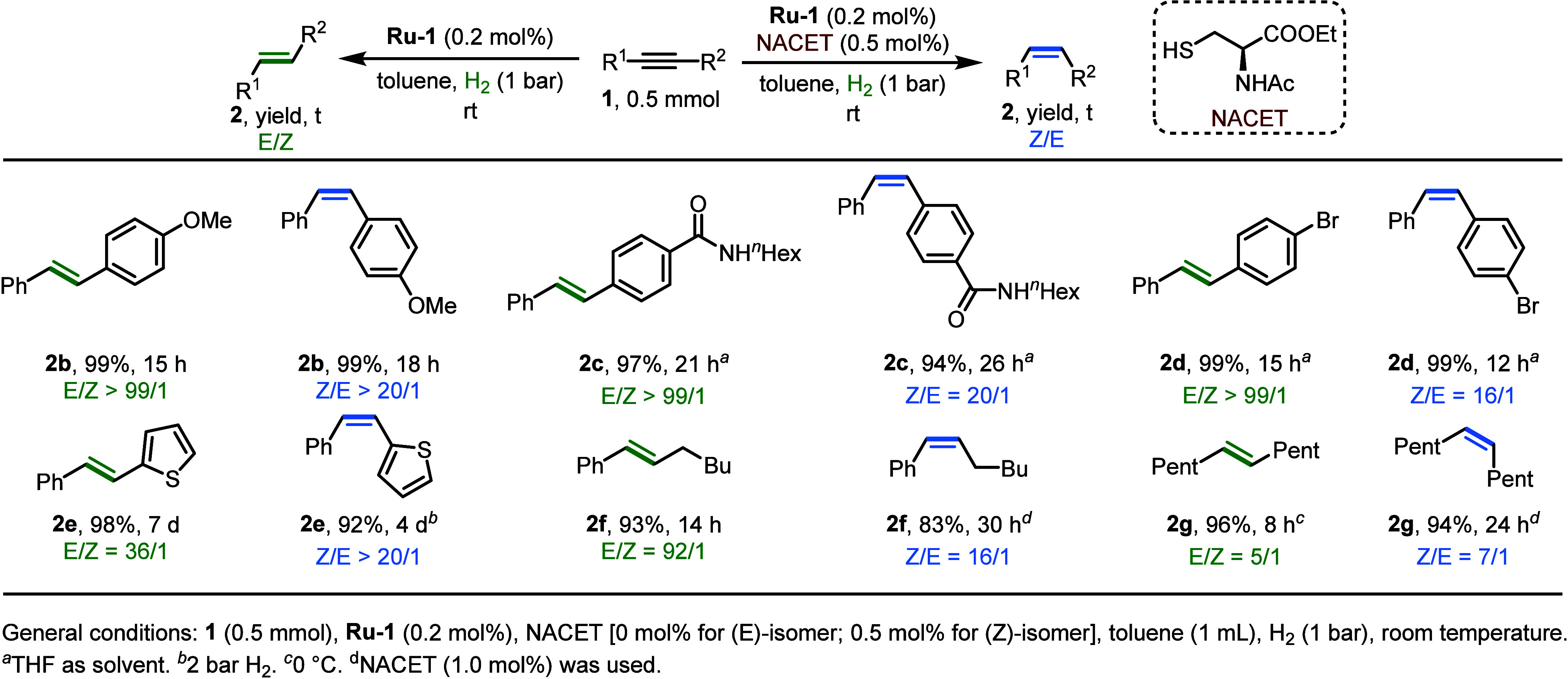
Representative selection of substrates investigated for thiol-controlled alkyne semihydrogenation.

**Scheme 6:**
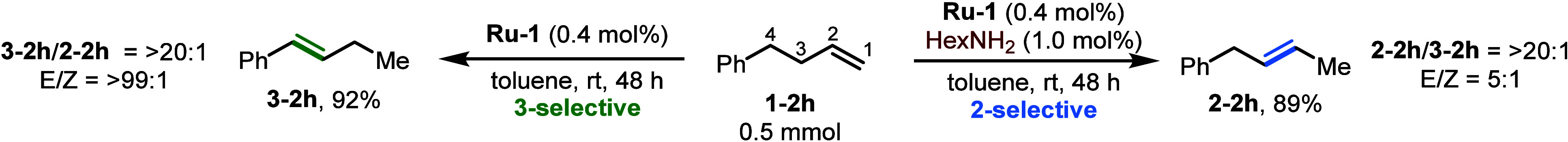
Amine-Controled Isomerization of an Alkene by Ru-1

The above results highlight the positive impact of the inhibition effects exerted by the thiol TCLs. While such inhibition does reduce the reaction rate, as reflected in longer reaction times (Scheme 5b,c) or lower TOFs (Scheme 4), it can enhance the selectivity of the catalytic process, as the thiol competes with the substrate for coordination to the ruthenium center. Capitalizing on this thiol-induced effect, we showed that the catalytic system involving NACET can effectively and selectively hydrogenate a series of arene- and alkyl-substituted alkynes (Figure 5). We have also demonstrated that this kind of inhibition effect can be achieved with an amine, instead of thiol, and have utilized it to control the isomerization of a representative terminal alkene, **1–2h** (Scheme 6).^3^

**Figure 6:**
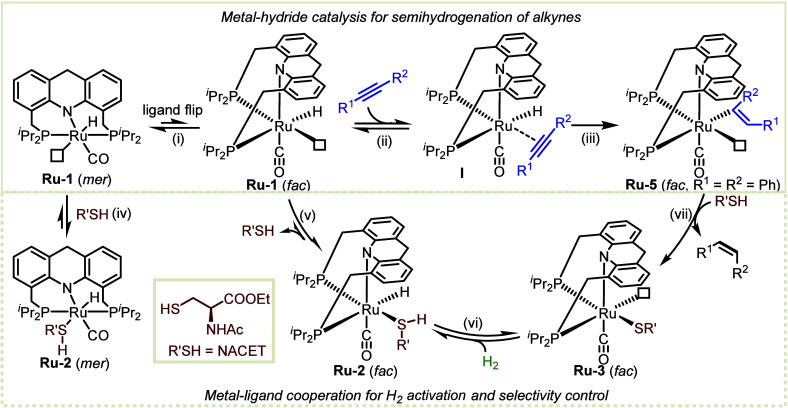
Proposed catalytic cycle for alkyne semihydrogenation by **Ru-1** with thiol as catalyst inhibitor.

The proposed mechanism of thiol-controlled alkyne semihydrogenation (Figure 6) was probed experimentally and computationally, allowing us to elucidate the role of the transient cooperative thiol ligand in this transformation. As described above, **Ru-2** forms *in situ* upon addition of thiol to **Ru-1** (steps iv and v). In this manner, the thiol serves as a reversible inhibitor, protecting the vacant site on the metal center of **Ru-1** from incoming substrate molecules. Nevertheless, the affinity of alkynes to the Ru(II) center is high enough to enable them to exchange with the thiol and eventually insert into the Ru–H bond (steps v, ii, and iii), thereby generating the alkenyl ruthenium species **Ru-5**. This latter complex can then react with the thiol to form **Ru-3** and release the (*Z*)-alkene product (step vii). The obtained (*Z*)-alkene typically has a much lower affinity for the metal center than does the alkyne or thiol, thereby preventing *Z*/*E* isomerization in the presence of thiol, as verified by control experiments (Figure 4). This, in turn, steers the reaction toward *cis*-semihydrogenation. Therefore, the transient nature of the ruthenium-bonded thiol, i.e., its ability to coordinate to the ruthenium center, and then dissociate from it, is responsible for the observed stereoselectivity of alkyne semihydrogenation. At the same time, the cooperative role of this thiol ligand, as reflected in the thiolate complex **Ru-3**, enables H_2_ activation by MLC to regenerate crucial Ru–H intermediates (steps vi and v), and is essential for ensuring catalytic turnover.

**Figure 7:**
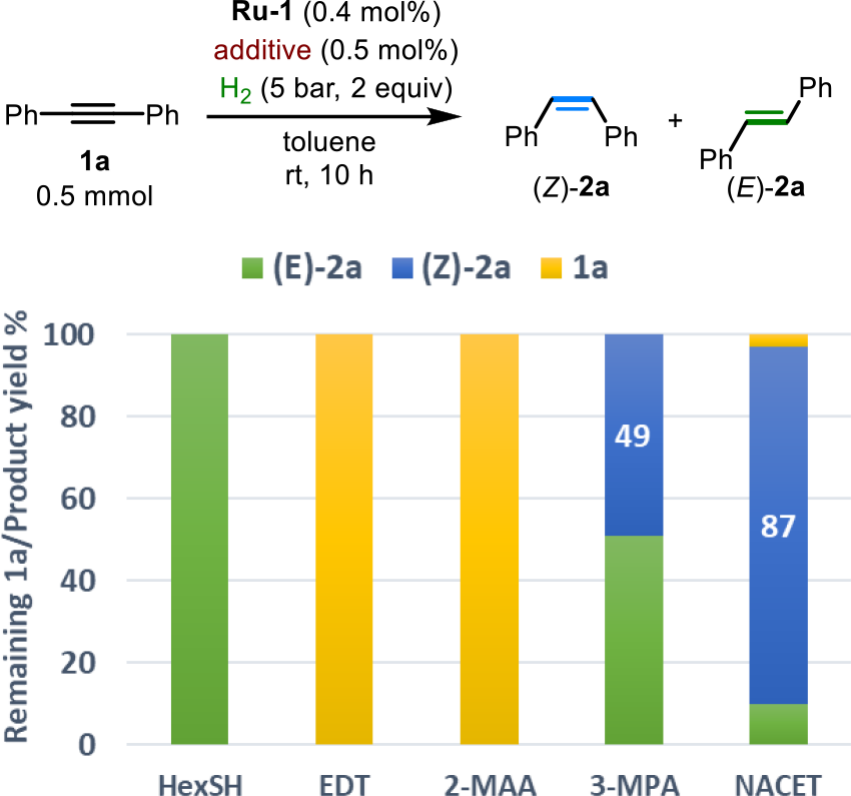
Screening of additives for alkyne semihydrogenation catalyzed by **Ru-1**. EDT, ethanedithiol; 2-MAA, *N*-decyl 2-mercaptoacetamide; 3-MPA, 3-mercaptopropionic acid.

Finally, it is important to note that different thiols may lead to diverging results, depending on the extent of their inhibitory effect. For instance, when **1a** was used as substrate, the addition of *N*-decyl 2-mercaptoacetamide (2-MAA) or ethanedithiol (EDT) prevented its hydrogenation altogether, with no alkyne conversion being observed (Figure 7). However, when HexSH was employed, (*E*)-**2a** was obtained as the only product. The distinct behavior of these thiols supports their involvement in the semihydrogenation reaction and further underscores the highly tunable nature of this type of TCL. Such catalytic tunability can therefore be achieved by simply changing the additive instead of synthesizing a new complex.

## Thiol-Induced Acceleration and Inhibition Effects Enable Hydrogenative Perdeuteration of C=C Bonds Using H_2_ and D_2_O^4^

**Scheme 7:**
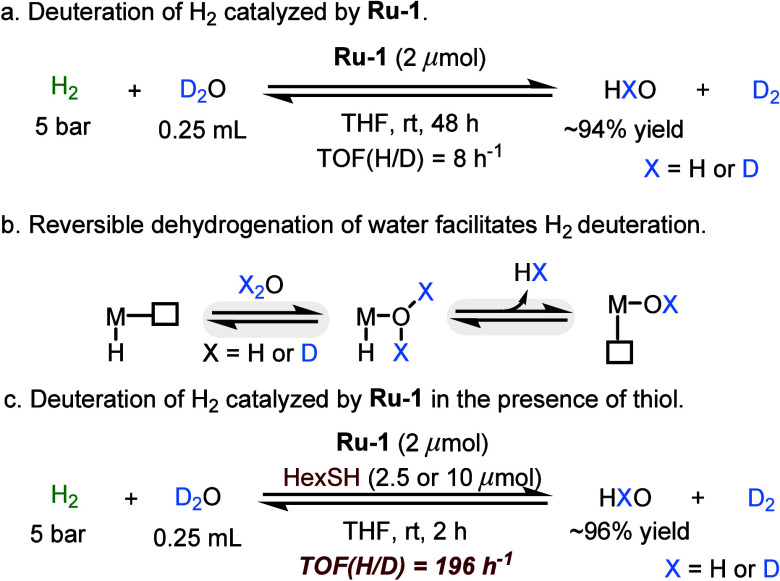
Acceleration Effect of Thiol in H/D Exchange between H_2_ and D_2_O Catalyzed by Ru-1

We have previously reported that **Ru-1** can catalyze H/D exchange between D_2_O and H_2_ to produce D_2_ (Scheme 7a).^4^ This H/D exchange occurs through reversible dehydrogenation of water at the ruthenium center,^37−40^ and the entire exchange process is driven by the excess of D_2_O (Scheme 7b). However, the deuterium labeling rate of this system was initially found to be slow, with a TOF of only 8 h^–1^. Nevertheless, adding HexSH in an amount equivalent to the catalyst increased the H/D exchange rate nearly 25-fold (Scheme 7c), in a manner reminiscent of the thiol-induced acceleration of methanol reforming. Notably, raising the amount of thiol to 5 equiv had no significant effect on this high H/D exchange rate, causing no substantial inhibition. This suggests that the reaction mechanism does not involve significant coordination of H_2_ or D_2_O to the metal center of **Ru-1**, which should become less coordinatively accessible as thiol concentration increases.

**Figure 8:**
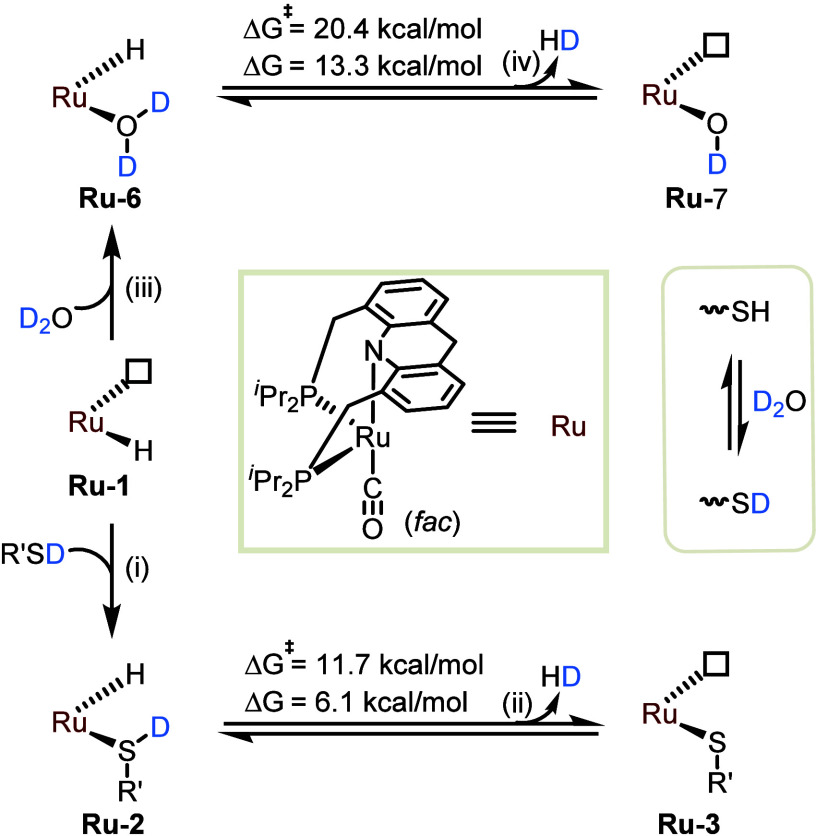
Elementary steps of H/D exchange between H_2_ and D_2_O catalyzed by **Ru-1** in the presence and absence of thiol, and respective calculated reaction energies.

In an attempt to rationalize the acceleration effect of thiol in this deuteration reaction, a plausible mechanism was proposed and studied computationally (Figure 8). It should be noted that both the free and coordinated thiol are relatively acidic, undergoing fast H/D exchange with the excess D_2_O, and serving as secondary deuterium sources. As discussed above, **Ru-1** combines with the externally added thiol to generate **Ru-2** (step i). This, in turn, is the catalytically active species that predominates in the presence of H_2_ throughout the duration of the isotopic exchange process. Importantly, our DFT results indicate that the extrusion of H_2_ from **Ru-2** to yield **Ru-3** (step ii) is both thermodynamically and kinetically much more favorable than the analogous reaction of the water adduct **Ru-6** to give the hydroxo complex **Ru-7** (step iv). These findings provide an explanation for the remarkable thiol-promoted acceleration of H_2_ deuteration.

**Figure 9:**
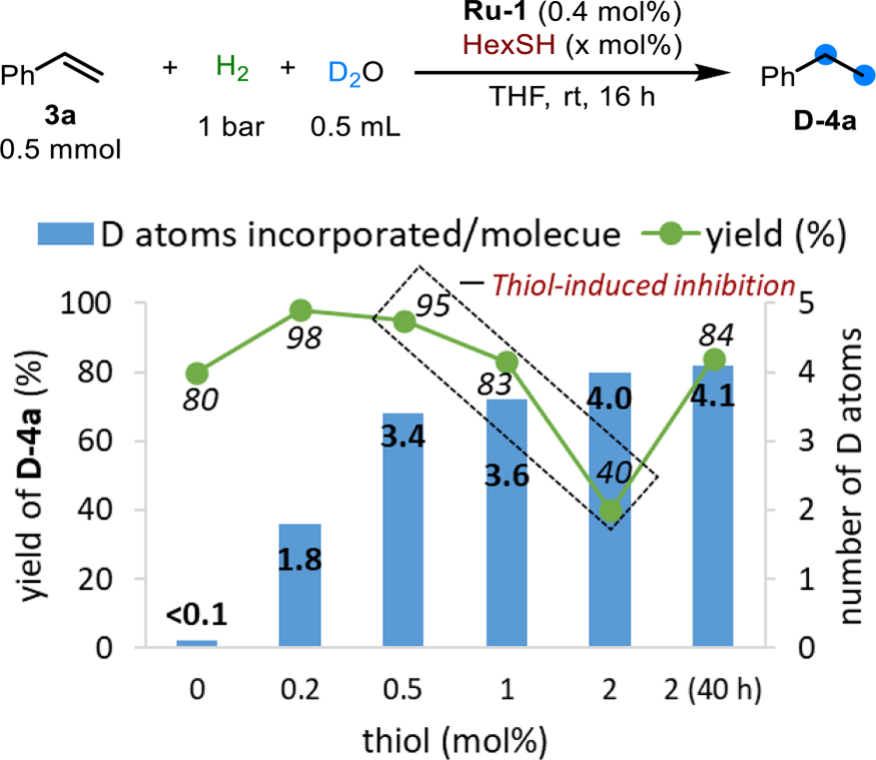
Effect of the amount of thiol on styrene C=C bond perdeuteration catalyzed by **Ru-1**, as reflected in D atom incorporation and perdeuterated product yield.

Capitalizing on this method of Ru-catalyzed, thiol-accelerated deuterium labeling of H_2_ with D_2_O, we were able to accomplish the single-step hydrogenative perdeuteration of alkenes without requiring the direct use of highly expensive D_2_. Using styrene (**3a**; Figure 9) as a model substrate, and applying **Ru-1** and HexSH in a ∼1:1 molar ratio, we observed the unexpected formation of overdeuterated ethylbenzene (**D-4a**), with 3.4 D atoms being incorporated per C=C bond, instead of only two D atoms, as would be expected for a typical deuterogenation reaction.^41^ In the absence of thiol, only negligible deuteration was detected in the product **4a** (<0.1 D per molecule), but increasing the amount of thiol enhanced this isotopic labeling, reaching as much as 4.0 D atoms per C=C bond with 5 equiv of thiol per catalyst, albeit at the expense of product yield. The drop in reaction rate, which is responsible for the lower yield, is consistent with thiol-induced inhibition, and indicates that the parent catalyst **Ru-1** is also directly involved in this hydrogenative alkene perdeuteration.

**Figure 10:**
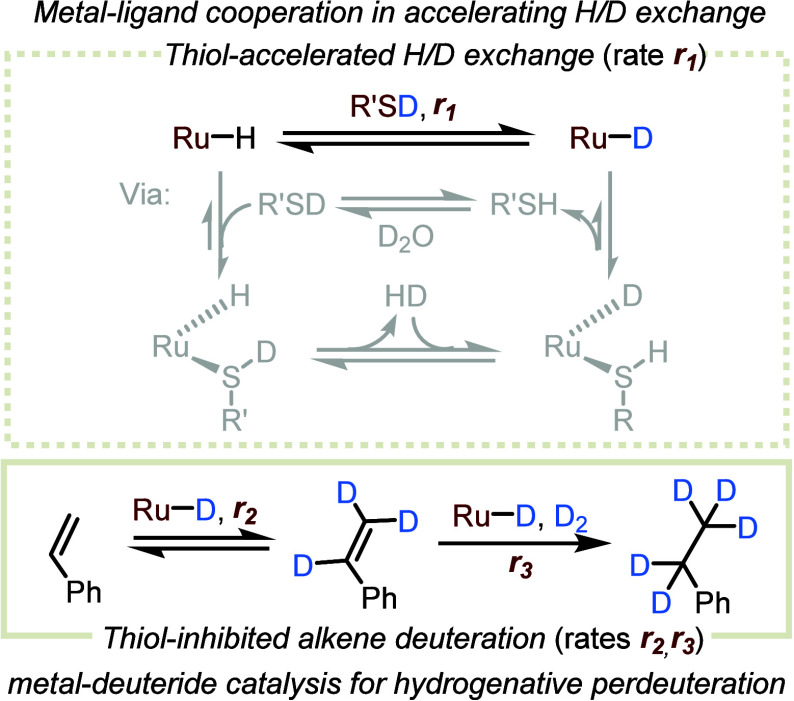
Acceleration and inhibition effects of thiol in the hydrogenative alkene perdeuteration catalyzed by **Ru-1**.

The mechanism by which perdeuterated **D-4a** is generated involves H/D exchange between D_2_O and **Ru-1** that is both mediated and enhanced by the thiol (rate *r*_1_; Figure 10), followed by the reversible insertion of styrene into the Ru–D bond (*r*_2_),^42^ which itself precedes the deuterogenation reaction (*r*_3_). A prerequisite for successful alkene perdeuteration is that both D_2_ and the Ru–D species are generated prior to the deuterogenation step (i.e., *r*_1_ > *r*_3_). The fact that we could achieve significant perdeuteration reflects the important role of thiol as a transient cooperative ligand. First, this thiol accelerates the formation of Ru–D species by cooperating with the ruthenium center. Second, it selectively inhibits the reactions of the alkene by competing with it for coordination to the metal center. These simultaneous acceleration and inhibition effects increase the rate difference between the generation of the alkene-activating Ru–D species and its subsequent reactions with the alkene, thereby achieving the selective inclusion of deuterium at the double bond, rather than protium.

**Figure 11:**
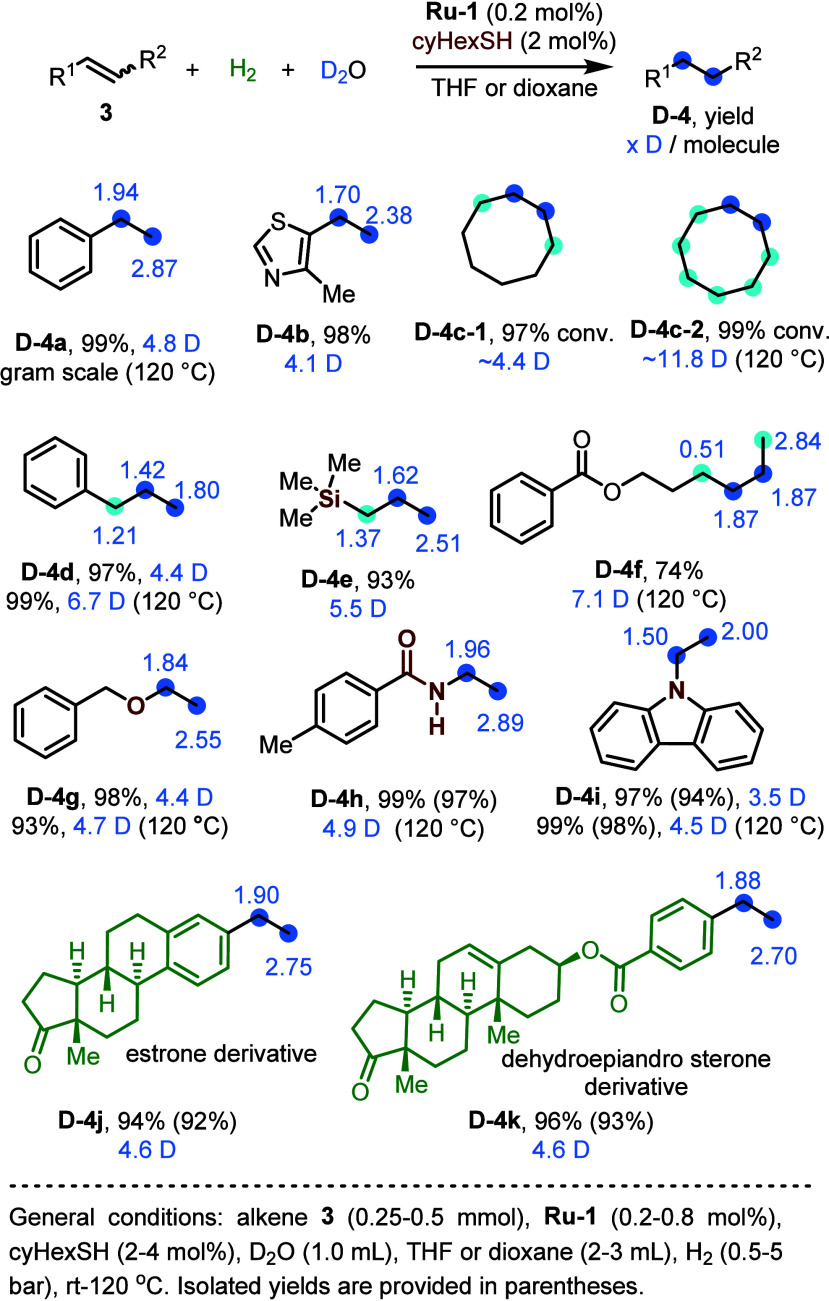
Representative examples of alkene substrates investigated for hydrogenative perdeuteration.

Our catalytic system was found to be highly effective in the hydrogenative perdeuteration of a variety of alkenes, using H_2_ and D_2_O as reagents (Figure 11). For most substrates, more than 4 D atoms could be incorporated per C=C bond, but increasing the reaction temperature and amount of D_2_O allowed us, in some instances, to reach as many as 4.9 D atoms per C=C bond. It is noteworthy that many current deuterated drugs, which have already been approved or are in clinical trials, often bear perdeuterated aliphatic groups at specific sites, alongside undeuterated aromatic rings.^43^ Our hydrogenative perdeuteration method provides a new way of constructing perdeuterated alkyl groups at particular sites, using readily available alkenes under mild conditions, and can contribute to the development of deuterated drug candidates.

## Transient Cooperative Thiol(ate)s as Products or Reactants^1,44,45^

**Scheme 8:**
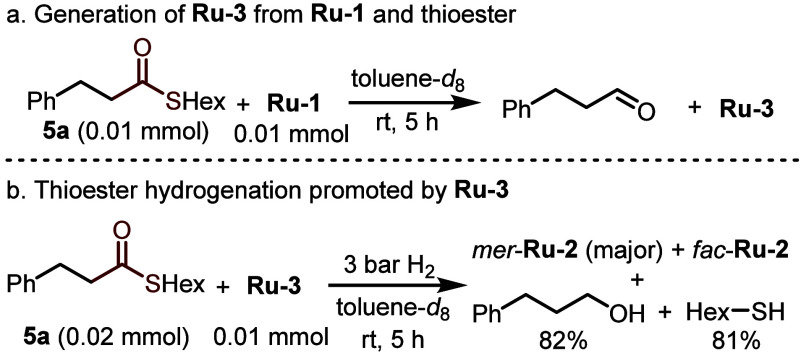
Stoichiometric Experiments toward Hydrogenation of Thioesters

**Figure 12:**
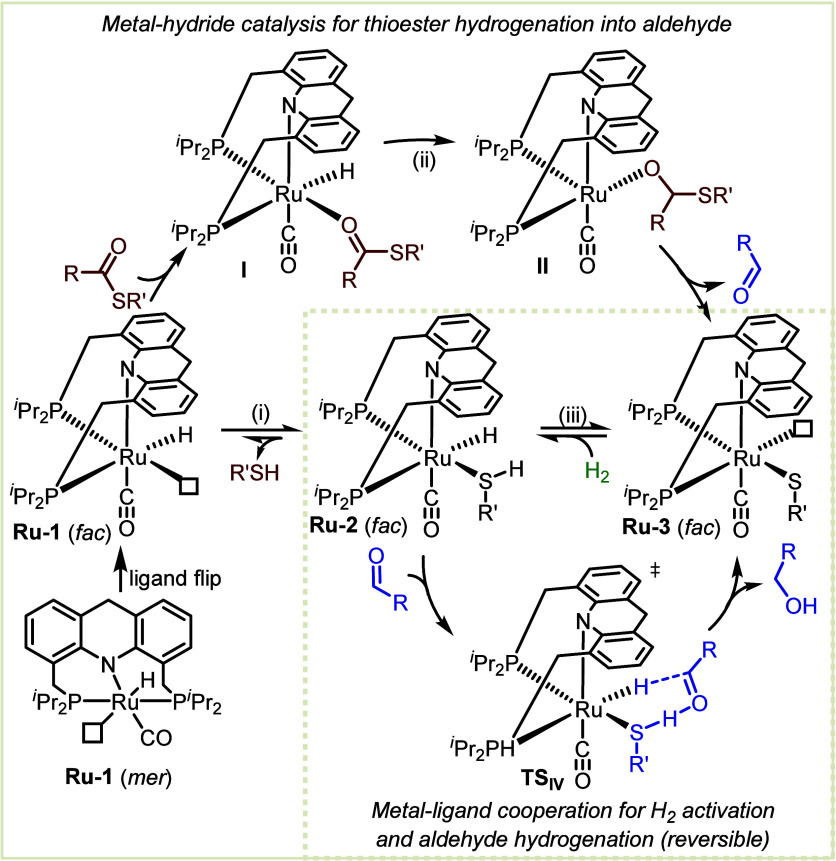
Proposed mechanism of thioester hydrogenation by **Ru-1**.

The realization that thiols can function as TCLs was borne out of our work on methanol reforming. However, in hindsight, their role as TCLs was already apparent in our earlier work involving thiols as products and reactants. We have previously reported that ruthenium thiolate complex **Ru-3** could be generated by the reaction of **Ru-1** with a thioester (**5a**; Scheme 8a), leading to the release of an aldehyde, and indicating that **Ru-1** can reduce thioesters.^44^ Interestingly, **Ru-3** itself was found to promote the hydrogenation of **5a** under H_2_ at room temperature, giving the corresponding alcohol and thiol, as well as complex **Ru-2** (Scheme 8b). When **Ru-1** was employed as catalyst for the same reaction, we noticed that its rate sharply decreased with time, and a thioester conversion of only 50% was reached after 36 h, improving to 70% after 5 d (Table 1, entries 1 and 2). Adding HexSH to the initial reaction mixture, in an amount equivalent to the substrate, impeded the reaction even further, with only 17% conversion being observed after 36 h (entry 3). This thiol-induced inhibition supports the direct involvement of **Ru-1** as catalyst in this hydrogenation reaction, although **Ru-2** was observed as the predominant complex during the reaction under hydrogen pressure. Notably, 3-phenylpropionaldehyde, an intermediate in the hydrogenation of **5a**, was fully hydrogenated by **Ru-1** even in the presence of HexSH in an amount equivalent to the aldehyde (entry 4). This indicates that the aldehyde is hydrogenated by **Ru-2** through an outer-sphere mechanism that does not involve aldehyde coordination (**TS**_**IV**_; Figure 12).

**Table 1:**
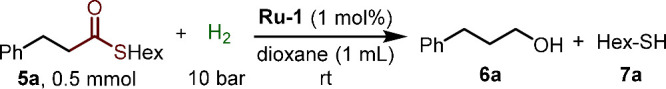
Thiol-Induced Inhibition in the Catalytic Hydrogenation of Thioesters by Ru-1

entry	HexSH (equiv)^a^	t	conversion **5a** (%)	yields **6a**/**7a** (%)
1	0	36 h	50	46/48
2	0	5 d	70	64/68
3	1	36 h	17	11/14
4^b^	1	36 h	92	81/-

a The cited amount of HexSH is in equivalents vs substrate.

b 3-Phenylpropionaldehyde (0.5 mmol) was used as substrate.

The above results support the involvement of both **Ru-1** and **Ru-2** in the catalytic thioester hydrogenation, as outlined in the proposed mechanism (Figure 12). Thus, after the *in situ* generation of **Ru-3**, its thiolate ligand cooperates with the ruthenium center to activate H_2_, thereby affording *fac-***Ru-2** (step iii). The latter can then release its thiol as product, with concomitant formation of **Ru-1**, which can subsequently reduce the thioester into aldehyde (steps i and ii). Moreover, **Ru-2** can directly catalyze the outer-sphere hydrogenation of the aldehyde intermediate, with the cooperative thiol ligand donating its proton, and the ruthenium center simultaneously donating its hydride (**TS**_**IV**_). This thioester hydrogenation mechanism was explored computationally, indicating that all kinetic barriers throughout the reaction profile are readily surmountable (≤25 kcal/mol).^45^

**Figure 13:**
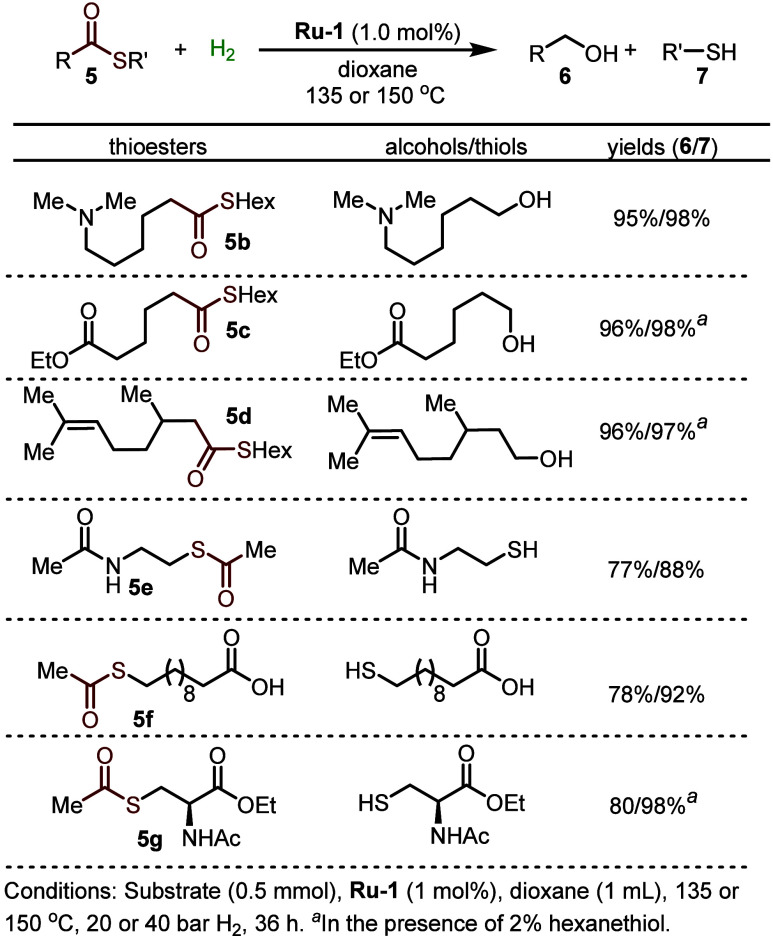
Hydrogenation of various thioesters.

**Figure 14:**
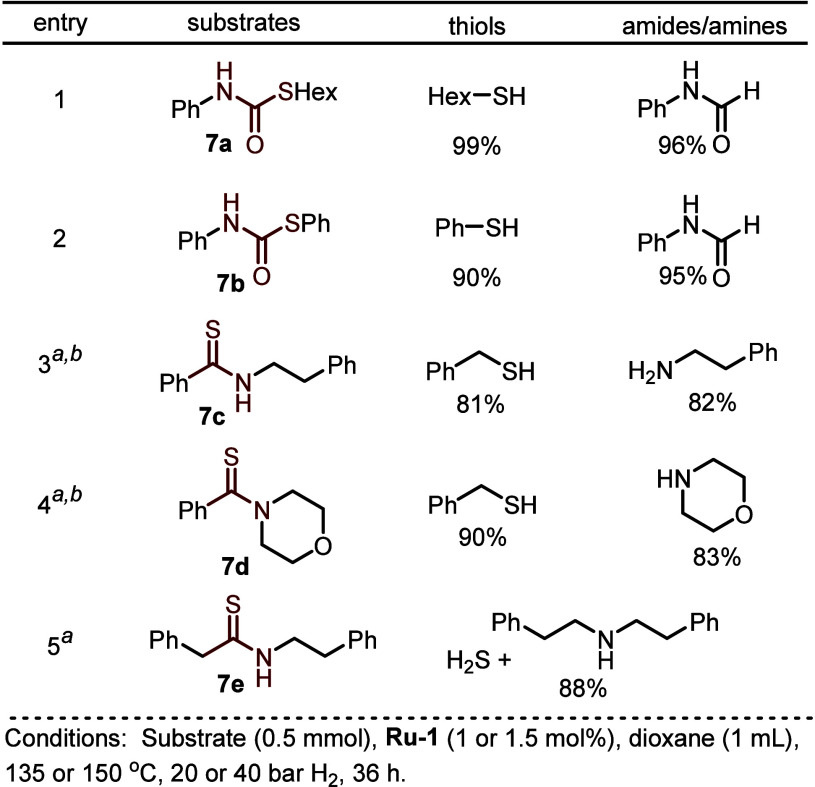
Hydrogenation of thiocarbamates and thioamides.

The inhibitory effect of the thiol can be overcome by increasing the temperature of the reaction mixture, thereby promoting thiol dissociation from the ruthenium center. Thus, full conversion of various thioesters into their respective alcohols and thiols was achieved, for the first time, by heating the reaction mixtures to 135 °C under H_2_, with no side reactions being observed (Figure 13).^44,46^ Our catalytic system was found to tolerate thioesters bearing an array of different functional groups, including amide, ester, carboxylic acid, and trisubstituted C=C bonds. It should be noted that ester groups remained untouched under the thioester hydrogenation conditions, even though esters can be fully hydrogenated by **Ru-1** in the absence of thiol.^47^ This implies that the excellent chemoselectivity of our thioester hydrogenation system benefits from the inhibition effect of the *in situ* generated thiol, which prevents other functional groups from interacting with the ruthenium center. Other sulfur compounds, such as thiocarbamates and thioamides, were also suitable substrates for this hydrogenation system (Figure 14).

**Figure 15:**
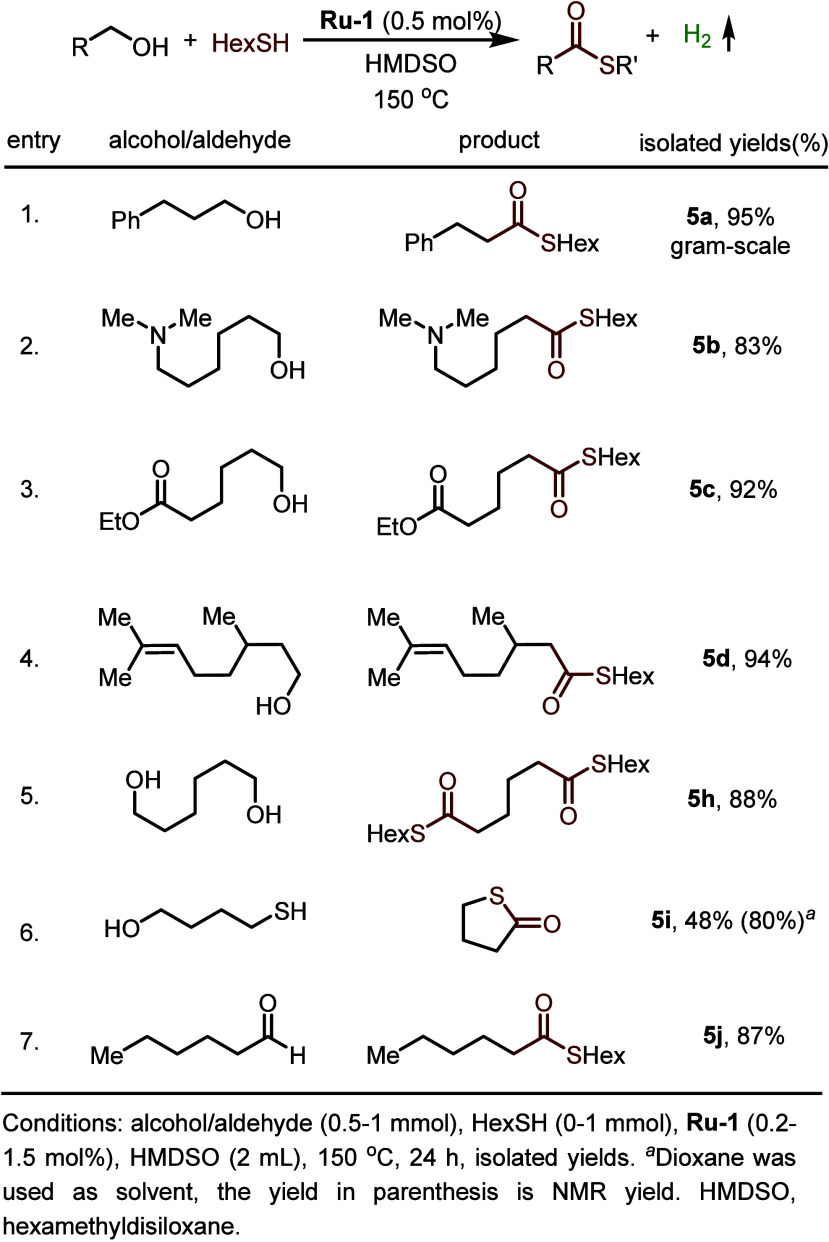
Formation of thioesters by dehydrogenative coupling of alcohols and HexSH.

Thioester hydrogenation by **Ru-1** is a reversible process. Thus, heating a 1:1 mixture of alcohol and thiol in the presence of this catalyst afforded the corresponding thioester (Figure 15), with liberation of H_2_.^1^ In this case, the *in situ* generated **Ru-3** not only transforms a given alcohol into an aldehyde intermediate through a cooperative outer-sphere dehydrogenation mechanism, but it also facilitates the conversion of this aldehyde into a thioester (Figure 12, with the catalytic cycle reversed).^45^ Thioesters play important roles in chemistry and biology, but their synthesis generally exhibits poor atom economy and generates copious waste.^48^ Our catalytic system provides an efficient, waste-free method of synthesizing a range of thioesters from the respective alcohols and thiols. Moreover, aldehydes can also be utilized as substrates, instead of alcohols, to give thioesters in excellent yields. These examples clearly demonstrate that a reactant or product can also serve as a TCL that participates in catalysis.

## Concluding Remarks

Over the past few years, we have developed and applied a new mode of metal–ligand cooperation involving thiols as *transient cooperative ligands* (TCLs). As described in this *Account*, these ligands can exert remarkable acceleration and inhibition effects in Ru-catalyzed (de)hydrogenation reactions, thereby enabling us to devise novel catalytic processes. This work highlights several advantageous aspects of TCLs, as follows:

- The participation of TCLs in catalytic reactions can significantly lower kinetic barriers in chemical bond activation, as evidenced by the observed acceleration effects, and in a manner similar to conventional MLC-based processes.
- The coordinative lability of a TCL allows the activity of the parent catalyst to be preserved to some extent, as demonstrated above by the transformations ascribed to **Ru-1**. Therefore, the presence of a TCL establishes a dual catalytic system, comprised of the parent complex and a TCL adduct thereof, each of which promotes its own characteristic reactions.
- The reversible coordination of a TCL to a catalytically active metal center can lead to competitive inhibition, as reflected in the abovedescribed inhibition effects of thiols. While this typically reduces the reaction rate, it can improve or even switch the selectivity.
- Unlike conventional MLC, wherein the cooperative ligand is permanently fixed to the metal center, a TCL is added *in situ* to a given catalytic complex. This makes MLC with TCLs highly tunable and easily implementable.

By drawing attention to the concept of TCL, and providing details on its practical application with thiols, we aim to expand the knowledge base concerning cooperative ligands and their impact on metal-based catalysis, and thus contribute to the development of new chemical and biomimetic catalytic systems. Furthermore, we believe that this concept can be applied to ligand types other than thiols, and can impart new functionalities on existing catalytic systems beyond those explored by us. For example, our preliminary results indicate that carboxylic acids can also serve as TCLs. While conventional MLC offers promising prospects for organic synthesis and sustainable processes, we trust that the incorporation of TCLs will constitute a significant advancement in this field of research, opening up previously unattainable avenues.
